# Comparison of the Effects of Nonprotein and Protein Nitrogen on Apoptosis and Autophagy of Rumen Epithelial Cells in Goats

**DOI:** 10.3390/ani10112079

**Published:** 2020-11-09

**Authors:** Zhiwei Kong, Chuanshe Zhou, Jinhe Kang, Zhiliang Tan

**Affiliations:** 1 CAS Key Laboratory for Agro-Ecological Processes in Subtropical Region, National Engineering Laboratory for Pollution Control and Waste Utilization in Livestock and Poultry Production, Hunan Provincial Key Laboratory of Animal Nutritional Physiology and Metabolic Process, Institute of Subtropical Agriculture, Chinese Academy of Sciences, Changsha 410125, China; kongzhiwei2013@126.com (Z.K.); kangjh@isa.ac.cn (J.K.); zltan@isa.ac.cn (Z.T.); 2 Key Laboratory of Optoelectronic Devices and Systems of Ministry of Education and Guangdong Province, College of Optoelectronic Engineering, Shenzhen University, Shenzhen 518060, China; 3 College of Food Engineering and Biotechnology, Han Shan Normal University, Chaozhou 521041, China; 4 Hunan Co-Innovation Center of Safety Animal Production, CICSAP, National Engineering Laboratory for Pollution Control and Waste Utilization in Livestock and Poultry Production, Changsha 410128, China

**Keywords:** ammonium chloride, methionine, apoptosis, autophagy

## Abstract

**Simple Summary:**

Proteins provide important raw materials for the self-renewal of cells in the gastrointestinal tract. Cell self-renewal is inseparable from the coordination between apoptosis and autophagy. Previous researchers have explored whether different nitrogen sources are linked to activation or inhibition of apoptosis/autophagy. However, information on the regulatory mechanism of apoptosis and autophagy induced by different nitrogen sources among cells in the gastrointestinal tract is limited. This study attempted to compare the effects of different nitrogen sources on apoptosis and autophagy of rumen epithelial cells in goats through molecular biology techniques such as flow cytometry, transmission electron microscopy, and fluorescence microscopy. On the basis of the obtained results, we found that autophagy had a less obvious ameliorative effect on ruminal epithelial cell apoptosis after treatment with protein nitrogen than after treatment with nonprotein nitrogen.

**Abstract:**

Protein nutrition is particularly important for the self-renewal processes of gastrointestinal epithelial cells. The self-renewal of cells is inseparable from the interaction between apoptosis and autophagy. However, there are few reports on the relationship between different nitrogen sources and apoptosis/autophagy. In this study, the relative protein expression of Bcl-2-associated X protein(Bax), caspase-3, and p62 was significantly higher (*p* < 0.05), while that of Bcl-xl, Bcl-2, Beclin1, and Microtuble-associated protein light chain 3 (LC3-II) was significantly lower (*p* < 0.05), in the NH_4_Cl group in comparison with the NH_4_Cl + 4-phenylbutyric acid (4PBA) group. In addition, the relative protein expression of Bax and caspase-3 was significantly higher (*p* < 0.05), while that of Bcl-2 and Bcl-xl was decreased significantly (*p* < 0.05), in the NH_4_Cl + 3-Methyladenine (3-MA) group and the methionine (Met) + 3-MA group in comparison with the NH_4_Cl group. Furthermore, the relative protein expression of Beclin1 and LC3B-II was significantly lower (*p* < 0.05), while that of p62 was significantly higher (*p* < 0.05), in the NH_4_Cl + Z-VAD-FMK group and the Met + Z-VAD-FMK group in comparison with the NH_4_Cl group. In conclusion, our results suggested that endoplasmic reticulum (ER) stress played a critical role in the crosstalk between apoptosis and autophagy induced by NH_4_Cl and Met. Autophagy had a more obvious ameliorative effect on ruminal epithelial cell apoptosis after treatment with nonprotein nitrogen than after treatment with protein nitrogen. These findings may reveal the molecular mechanism of apoptosis and autophagy induced by nonprotein nitrogen and protein nitrogen.

## 1. Introduction

Apoptosis, which plays a pivotal role in tissue homeostasis by eliminating damaged or unnecessary cells in response to various stimuli, is the main cause of the shedding of normal intestinal mucosal epithelial cells and is closely related to the regeneration and repair of gastrointestinal cells [1]. In addition, exfoliation of mucosal epithelial cells during the renewal of gastrointestinal tissue is the largest contributor to the metabolism of fecal nitrogen in ruminants [2]. Autophagy maintains cellular homeostasis to promote cell survival by removing long-lived proteins and damaged organelles under conditions of starvation or other stresses. In many cases, autophagy negatively regulates apoptosis, whereas apoptosis activation blocks autophagy. Apoptosis and autophagy can be induced by modulating the ROS (oxygen free radical)/MAPK (mitogen-activated protein kinase) [3], PI3K(phosphatidylinositol 3-kinase)/Akt(serine-threonine kinase)/mTOR(mammalian target of rapamyoin) [4], and JNK(Jun N-terminal kinase) and mTOR [5] pathways.

Several amino acids (AAs) are associated with apoptosis/autophagy activation or inhibition. Currently, it is recognized that these AAs participate in the mTORC1 and GCN2/eIF2(Protein kinase GCN2/Eukaryotic initiation factor 2) pathways, regulating protein translation and controlling cell AA requirements by simultaneously regulating autophagy-dependent catabolism [6]. For example, methionine (Met) can reduce apoptosis of lymphocytes in peripheral blood and increase the Bcl-2(B-cell lymphoma-2 protein)/Bax(Bcl-associated protein X) ratio [7]. Researchers have found that exogenous Met also inhibits autophagy in liver cells treated via in vitro perfusion [8]. Additionally, studies have shown that Met regulates autophagy in BMECs(bovine mammary epithelial cells) through the PI3K/mTOR signaling pathway [9]. Notably, it has been reported that nonprotein nitrogen (NPN), such as that in ammonium chloride (NH_4_Cl), induces apoptosis by increasing oxidative damage and Cyt C(Cytochrome C) release [10,11,12]. When ammonia is present in excess, it can cause alkaline chemical burns of the mucosa [13]. Furthermore, NH_4_Cl can inhibit autophagy through the p38 MAPK pathway [14,15].

AAs, the most basic units used for metabolic activity in gastrointestinal tissue, are utilized by epithelial cells for protein synthesis to meet the growth, reproduction and metabolism needs of cells. NH_4_Cl is widely used as a source of NPN in livestock production. However, there have been few reports on the differences between apoptosis and autophagy induced by different nitrogen sources. Therefore, this study compared the effects of different nitrogen sources on apoptosis and autophagy in goat rumen epithelial cells. The findings will help elucidate the mechanisms of self-renewal in gastrointestinal epithelial cells and endogenous nitrogen secretion in ruminants.

## 2. Materials and Methods

### 2.1. Culture and Treatment of Primary Ruminal Epithelial Cells (PRECs)

The rumen tissues of healthy black goats were repeatedly rinsed with normal saline and then placed into phosphate-buffered saline (PBS) containing streptomycin, amphotericin B, and gentamicin for storage until use. Isolation, purification, identification, and culture of the PRECs were conducted on the basis of previously described methods [16]. Briefly, the epithelial layer of rumen tissue was separated from the muscle layer, added to a digestive solution containing 2.5% trypsin, and incubated for 5 min at 37 °C. The epithelial tissues were digested 5 times; the supernatant was discarded after the first 2 digestions, while the supernatant and precipitates were saved after the final 3 digestions. Then, the cells were resuspended in complete medium, inoculated into culture dishes, and cultured in an incubator at 37 °C. The medium was changed every 2 days. The purified cells were cultured in Dulbecco’s modified Eagle’s medium: Nutrient Mixture F-12 (DMEM/F12; Invitrogen, Carlsbad, CA, USA) containing 5% fetal bovine serum (FBS; Gibco, Grand Island, NE, USA), penicillin (100 units/mL, Invitrogen, Carlsbad, CA, USA)–streptomycin (0.1 mg/mL, Invitrogen, Carlsbad, CA, USA), gentamicin (25 g/mL, Invitrogen, Carlsbad, CA, USA), and amphotericin B (2.5 g/mL, Invitrogen, Carlsbad, CA, USA) at 37 °C in a cell incubator containing 5% CO_2_ at a constant temperature and humidity. Met-free medium was prepared on the basis of the DMEM/F12 formula. NH_4_Cl was freshly diluted in Met-free medium and added to the culture medium before treatment on the basis of the DMEM/F12 formula. When the PRECs reached 70% confluency, the cells were starved for 6 h in Met-free medium, and then cultured with the fresh medium containing 5 mM Met (control group) or 5 mM NH_4_Cl (treatment group) for 36 h (proliferation stage).

### 2.2. Morphological Detection

The fluorochrome Hoechst 33,342 (14,533, Sigma, MO, USA) was applied to detect chromatin condensation as previously described [17], and 4-phenylbutyric acid (4PBA) was added to confirm the role of endoplasmic reticulum (ER) stress in apoptosis. Briefly, after treatment for 36 h with 5 mM Met or NH_4_Cl, PRECs were fixed for 30 min at 4 °C with 4% paraformaldehyde. Then, the cells were treated with PBS (5 min per wash). The cells were then incubated with Hoechst 33,342 (8 μg/mL) in the dark for 30 min at room temperature. Finally, the cells were observed under a fluorescence microscope to determine the ratio of apoptotic nuclei. 

### 2.3. Monodansylcadaverine (MDC) Staining Analysis

MDC (KGATG002, KeyGEN, Nanjing, China) was used as a tracer for autophagy [18]. After PRECs were treated for 36 h, MDC (50 μm) was added, and the cells were incubated in the dark for 20 min at a constant temperature of 37 °C in a 5% CO_2_ incubator. The morphological changes related to autophagy were observed by fluorescence microscopy.

### 2.4. Apoptosis Detection by Flow Cytometry

PRECs were inoculated into 6-well plates at a density of 1 × 10^6^ cells per well and cultured with Met or NH_4_Cl. The cells were collected, centrifuged at 1500 rpm for 5 min, and then resuspended in 500 μL of binding buffer. Finally, the cells were incubated with 5 μL of annexin V-FITC(Fluorescein Isothiocyanate, Nanjing, Jiangsu, China) and 5 μL of propidium iodide (PI) (Annexin V-FITC/PI Apoptosis Detection Kit, KGA108, KeyGEN) in the dark for 15 min at room temperature. Flow cytometry (BD FACScalibur, San Jose, CA, USA) was used to analyze the apoptosis rate.

### 2.5. Intracellular ROS Detection

Cells were collected and exposed to pre-warmed PBS containing DCFHDA(2,7-Dichlorodi -hydrofluorescein diacetate) (10 μM) for 1 h at 37 °C. Then, the cells were washed and resuspended in PBS. Finally, a flow cytometer was used to detect the fluorescence intensities of cells at an excitation/emission wavelength of 488/525 nm. FlowJo software (FlowJo, Version 7.6.1; TreeStar, Ashland, OR, USA) was applied to analyze the fluorescence intensities data. 

### 2.6. Detection of Mitochondrial Membrane Potential (MMP) 

A JC-1 assay kit (MCE3520-43-2, Shanghai, China) was used to measure the MMP [17]. Cells were collected, washed with PBS, and then incubated with JC-1 (10 μg/mL) in the dark for 20 min at 37 °C. Finally, a flow cytometer was used to detect the fluorescence intensity of the cells (BD FACScalibur).

### 2.7. Transmission Electron Microscopy (TEM) Analysis

After treatment with 5 mM Met or NH_4_Cl for 36 h, PRECs were fixed successively with 1% osmic acid fixation solution and 2.5% glutaraldehyde for more than 2 h. Then, the cells were washed with phosphate buffer (0.1 mM) and dehydrated with a gradually increasing concentrations of acetone. After this, they were coated with epoxy resin, the cell samples were sliced. A transmission electron microscope (JEM-2000EX, JEOL Co, Tokyo, Japan) was applied to collect the electron images [17].

### 2.8. Tandem mCherry-EGFP-LC3 Immunofluorescence 

Cells were transfected with a rAd-mCherry-GFP-LC3B((Green fluorescent protein-microtubule associated protein-2) plasmid using Lipofectamine 2000 (Invitrogen, Carlsbad, CA, USA) according to the manufacturer’s instructions. At 24 h after transfection, the cells were treated with DMEM/F12 with 10% FBS and penicillin–streptomycin (Corning, 30-001-CI) for another 36 h. The fluorescence of rAd-mCherry-GFP-LC3B was observed. The autophagosomes (green dots) and autolysosomes (red dots) in each cell were counted under fluorescence microscopy (Leica DM2500). The rAd-mCherry-GFP-LC3B plasmid was purchased from Honor Gene (HG-rAd022867, Changsha, Hunan, China).

### 2.9. RT-PCR

After treatment for 36 h with 5 mM Met or NH_4_Cl, total RNA was extracted using AccuZol Total RNA Extraction Reagent (Bioneer, Daejeon, Korea) on the basis of the manufacturer’s instructions [19]. Then, DNaseI (Thermo Scientific, Waltham, MA, USA) was used to eliminate genomic DNA, and a Nano Drop 2000 (Thermo Scientific, Waltham, MA, USA) was used to evaluate the RNA quality and quantity. Then, a Prime Script RT Reagent Kit (Takara, Dalian, China) was applied to reverse-transcribe the RNA into cDNA(complementary Deoxyribonucleic acid). The 2^−ΔΔCT^ method was used to determine the relative mRNA expression levels [20]. The details of the primers used for the various genes are shown in Table 1. 

### 2.10. Western Blot Analysis

Protein isolation and Western blot analysis were conducted on the basis of previously described methods [21,22]. Briefly, each sample and a pre-stained standard were electrophoretically separated on 10% SDS-polyacrylamide gels (Bio-Rad Laboratories, Berkeley, CA, USA). The separated proteins were transferred to polyvinylidene fluoride (PVDF) membranes (Bio-Rad Laboratory) at a constant current 200 mA for 70 min. The PVDF membranes were incubated overnight in a solution containing primary antibodies (see Table 2) at 4 °C, washed 5 times, and then incubated with secondary antibodies (1:6000, Proteintech) at room temperature for 2 h while protected from light. An Alpha Imager 2200 digital imaging system (Digital Imaging System, Kirchheim, Germany) was used to obtain and analyze images.

### 2.11. Statistical Analysis

SPSS Statistical Software was used to analyze the data obtained. ROS levels, apoptosis rates, fluorescence intensity, autophagosome numbers, relative apoptotic and autophagic gene mRNA expression, LC3 spot numbers, protein abundance, and ER cavity size were analyzed using one-way ANOVA followed by Tukey–Kramer multiple comparisons tests. The data are shown as the mean ± SD.

## 3. Results

### 3.1. Apoptosis and Autophagy Induction by NH_4_Cl and Met in PRECs

**Figure 1:**
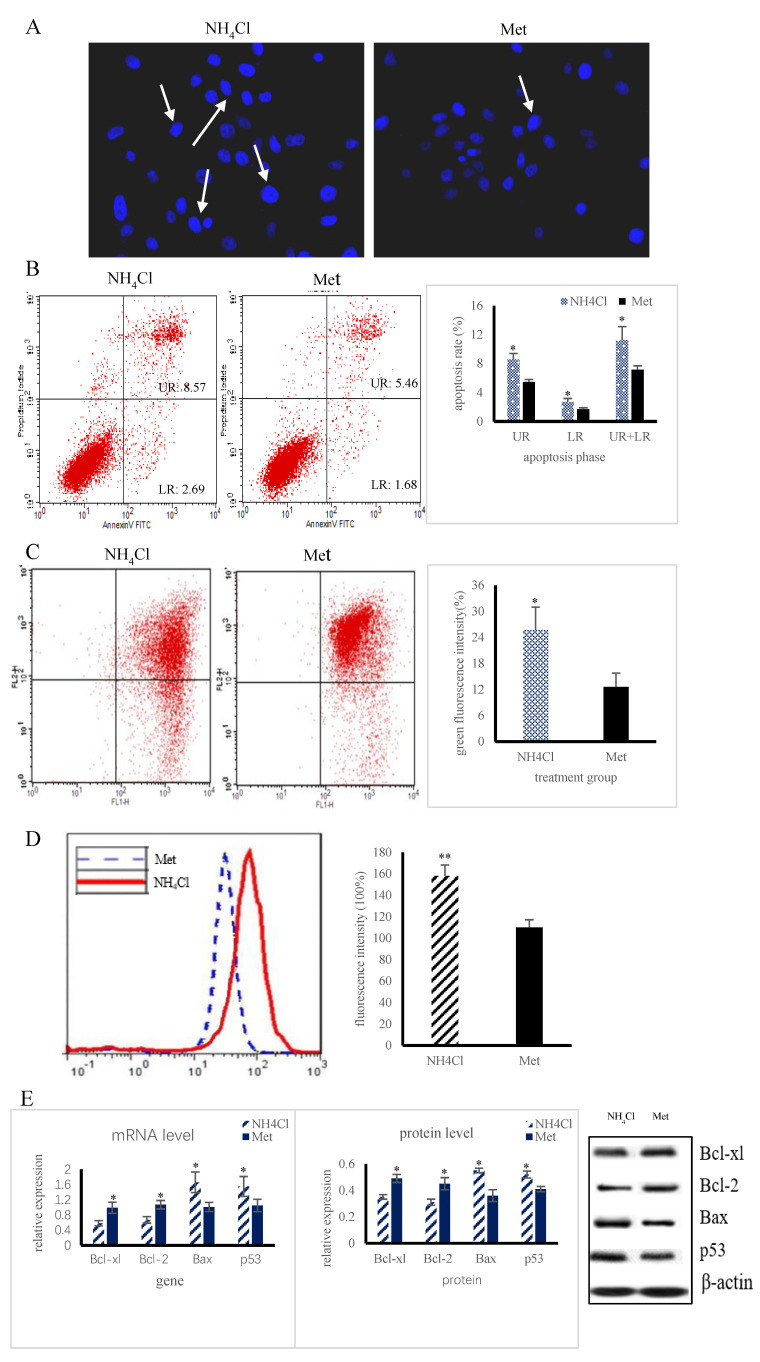
Changes of cell apoptosis after treatment with different nitrogen sources (*n* = 3). (**A**) Hoechst 33,342 staining in primary ruminal epithelial cells (PRECs) after 36 h treatment with 5 mM NH_4_Cl and methionine (Met). (**B**) Apoptosis rate percentage under NH_4_Cl and Met detected by flow cytometry. (**C**) Changes of mitochondrial membrane potential (MMP) under NH_4_Cl and Met detected by flow cytometry. (**D**) A histogram shows DCF fluorescence in the PRECs. In the histogram, the rise of ROS levels is shown as a shift to the right and a reverse decline to the left. (**E**) Expression of apoptotic genes and proteins under NH_4_Cl and Met detected by RT-PCR and Western blot. The data are expressed as mean ± SD. Compared with the control, * *p* < 0.05 and ** *p* < 0.01 show that the difference is statistically significant.

The PRECs in the Met group showed normal shapes with round, intact nuclei, whereas the cells treated with NH_4_Cl showed nuclear shrinkage, chromatin condensation, or fragmentation (Figure 1A). In addition, the apoptosis rate was significantly higher (*p* < 0.05) in the NH_4_Cl supplementation group than in the Met group (Figure 1B). The MMP change in the NH_4_Cl group was significantly larger (*p* < 0.05) than that in the Met group (Figure 1C). Cytoplasmic ROS levels were higher (*p* < 0.05) in NH_4_Cl-treated cells than in Met-treated cells (Figure 1D). Compared with Met addition, NH_4_Cl addition significantly increased (*p* < 0.05) the expression of Bax and caspase-3 at the mRNA and protein levels, while significantly decreasing (*p* < 0.05) the expression of Bcl-xl and Bcl-2 (Figure 1E). 

**Figure 2:**
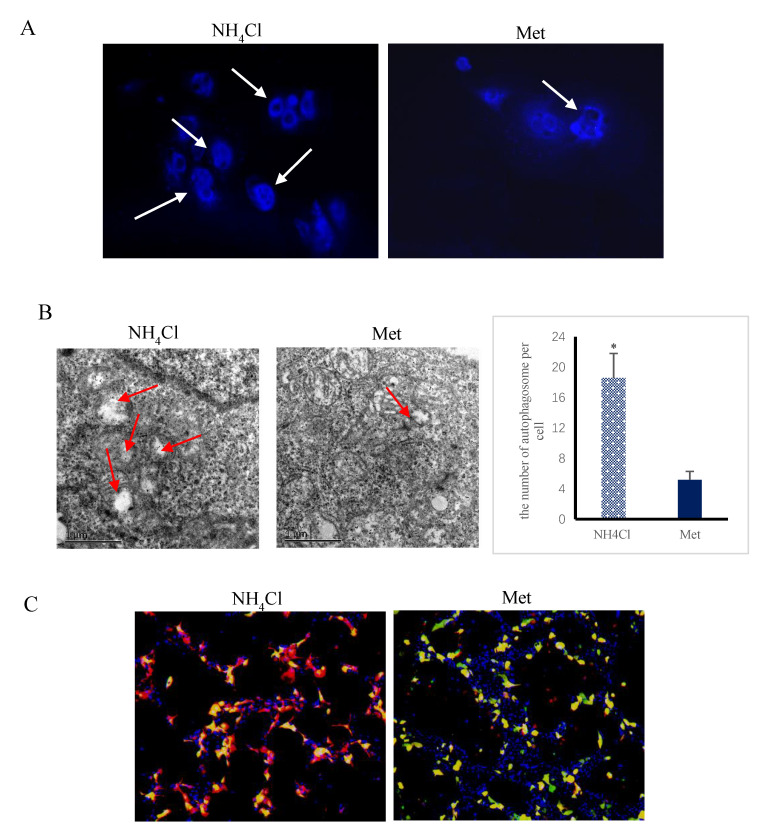

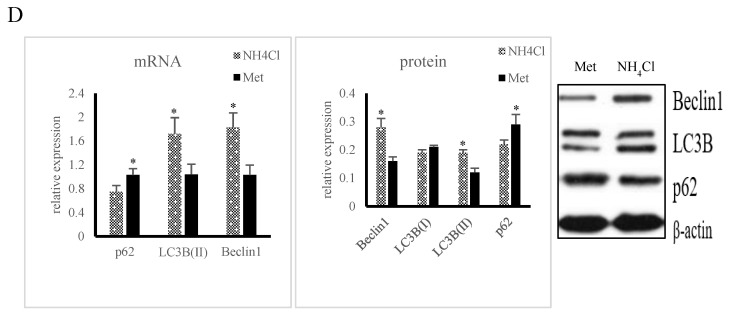
Changes of cell autophagy after treatment with NH_4_Cl and Met (*n* = 3). (**A**) Autophagic morphological changes were obtained on the basis of the results of monodansylcadaverine (MDC) staining; images were viewed under a 200× fluorescence microscope. (**B**) The cells were fixed and analyzed by transmission electron microscopy (40,000×). The number of autophagosomes per cell were determined from at least 15 cells in each group. The data are presented as mean ± SD. Compared with the control, * *p* < 0.05 shows the difference is statistically significant. (**C**) Immunofluorescence image of endogenous LC3 in PREC culture treated with NH_4_Cl and Met. Green dots represent autophagosomes, red dots represent autolysosomes, blue represents the staining with the Hoechst 33,342, and yellow is a merging of red and green. (**D**) Expression of autophagic genes and proteins under NH_4_Cl and Met detected by RT-PCR and Western blot.

The cytoplasmic fluorescence intensity was higher (*p* < 0.05) in NH_4_Cl-treated cells than in Met-treated cells (Figure 2A). In addition, the number of autophagosomes per cell was higher (*p* < 0.05) in NH_4_Cl-treated cells than in Met-treated cells (Figure 2B). After GFP-LC3 plasmid transfection, there were more LC3 spots in the NH_4_Cl group (*p* < 0.05) than in the Met group (Figure 2C). The mRNA and protein expression levels of LC3-II and Beclin1 were higher (*p* < 0.05), while those of p62 were lower (*p* < 0.05) in NH_4_Cl-treated cells in comparison with Met-treated cells (Figure 2D).

### 3.2. ER Stress Induction by NH_4_Cl and Met in PRECs

**Figure 3:**
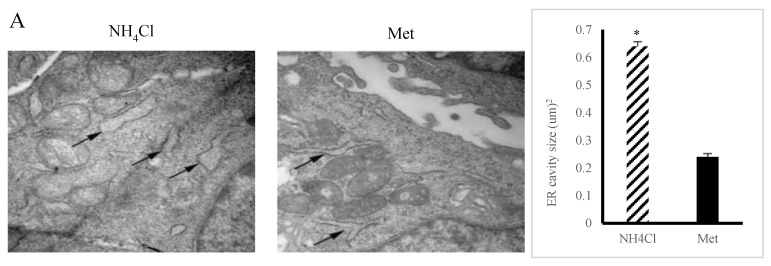

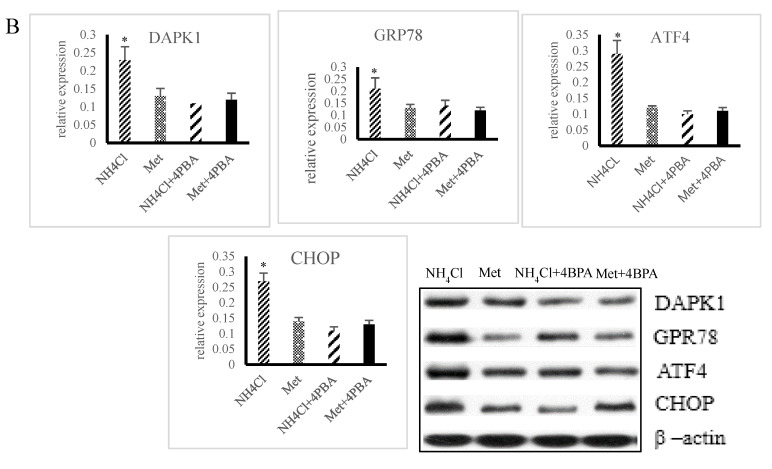
Endoplasmic reticulum (ER) stress induced by nonprotein nitrogen (NH_4_Cl) and protein nitrogen (Met) sources in PRECs. (**A**) Morphological changes of ER in nonprotein nitrogen (NH_4_Cl) and protein nitrogen (Met) were obtained by transmission electron microscopy. The images were viewed under a 40,000× transmission electron microscope. (**B**) The expression of ER stress-related proteins was conducted by Western blot. Each bar shows mean ± SD. “*” means there is a significant difference.

The expansion area of the ER cavity was larger (*p* < 0.05) in NH_4_Cl-treated cells than in Met-treated cells (Figure 3A). Concurrently, the protein expression levels of DAPK1(Death-associated Protein Kinase 1), GRP78 (glucose-regulated protein 78), ATF4 (activating transcription factor 4), and CHOP (C/EBP homoiogousprotein) were higher (*p* < 0.05) in the NH_4_Cl group than in the Met group (Figure 3B), while the protein expression levels of DAPK1, GRP78, ATF4, and CHOP were lower (*p* < 0.05) in NH_4_Cl + 4PBA-treated cells than in NH_4_Cl-treated cells (Figure 3B). There were no differences (*p* > 0.05) in the protein expression levels of DAPK1, GRP78, ATF4, and CHOP between the Met group (*p* > 0.05) and the NH_4_Cl + 4PBA group.

### 3.3. Effects of ER Stress on Apoptosis Induced by NH_4_Cl and Met in PRECs

**Figure 4:**
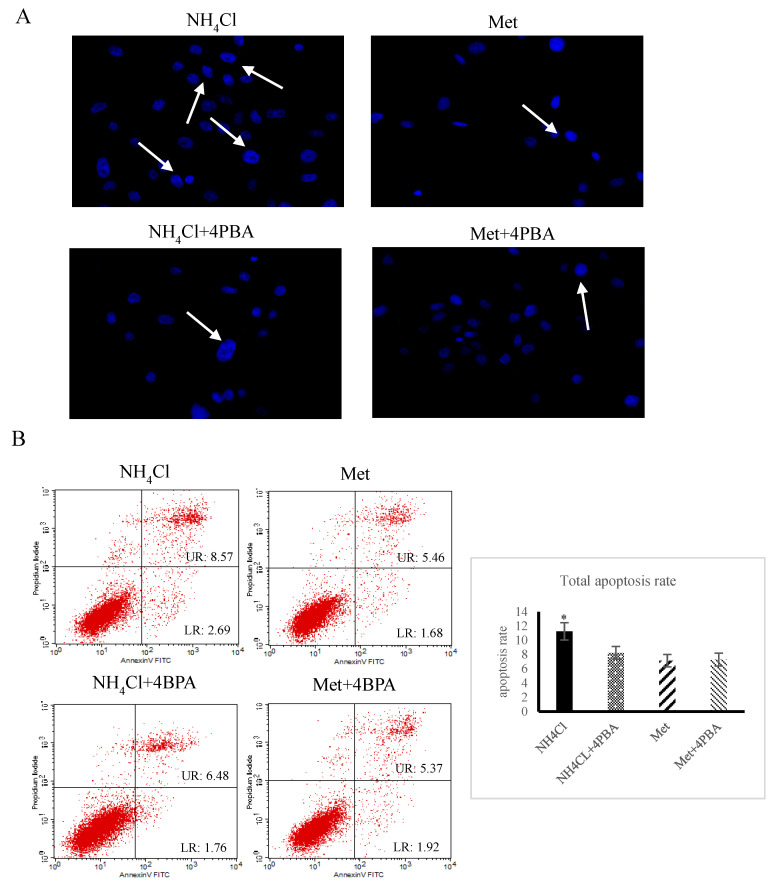

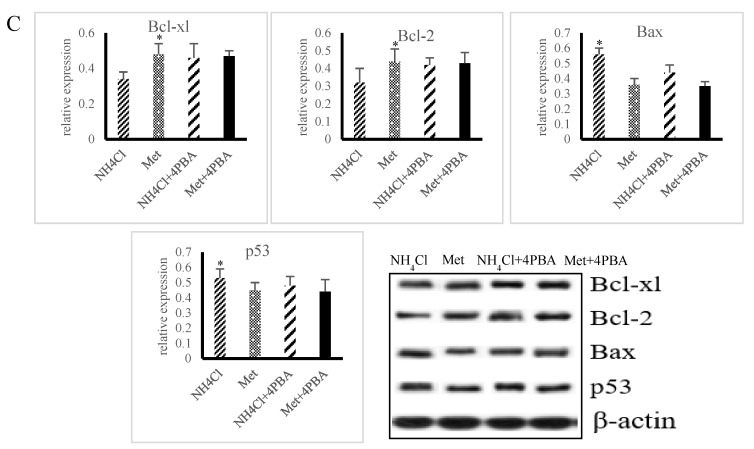
The effects of ER stress on NH_4_Cl and Met-induced apoptosis in PRECs. (**A**) Apoptotic morphological changes were obtained in PRECs on the basis of Hoechst 33,342 staining. The images were viewed under a 200× fluorescence microscope. (**B**) The effect of 4-phenylbutyric acid (4PBA) on apoptosis rate induced by NH_4_Cl and Met. (**C**) Western blot was conducted to confirm the role of 4PBA in apoptosis induced by inorganic nitrogen (NH_4_Cl) and organic nitrogen (Met). Each bar shows mean ± SD.

The cytoplasmic fluorescence intensity was higher (*p* < 0.05) in NH_4_Cl-treated cells than in Met-treated cells (Figure 4A), and no significant differences were observed between Met-treated cells and NH_4_Cl + 4BPA treated cells (*p* > 0.05). There were significantly more (*p* < 0.05) apoptotic cells in the NH_4_Cl group than in the NH_4_Cl + 4PBA group, while no significant difference (*p* > 0.05) was observed between the Met group and the Met + 4PBA group (*p* > 0.05). The number of apoptotic cells in the Met + 4PBA group was not significantly different from that in the NH_4_Cl + 4PBA group (*p* > 0.05) (Figure 4B). As shown in Figure 4C, compared to cells treated with Met only, cells treated with NH_4_Cl + 4PBA exhibited significantly higher relative protein expression of Bax and caspase-3 (*p* < 0.05), and Bcl-xl and Bcl-2 protein expression was significantly lower (*p* < 0.05) in the NH_4_C group than in the NH_4_Cl + 4PBA group. There were no significant differences in the expression of apoptotic proteins in the Met + 4PBA group compared with the Met group (*p* > 0.05) (Figure 4C).

### 3.4. Effects of ER Stress on Autophagy Induced by NH_4_Cl and Met in PRECs

**Figure 5:**
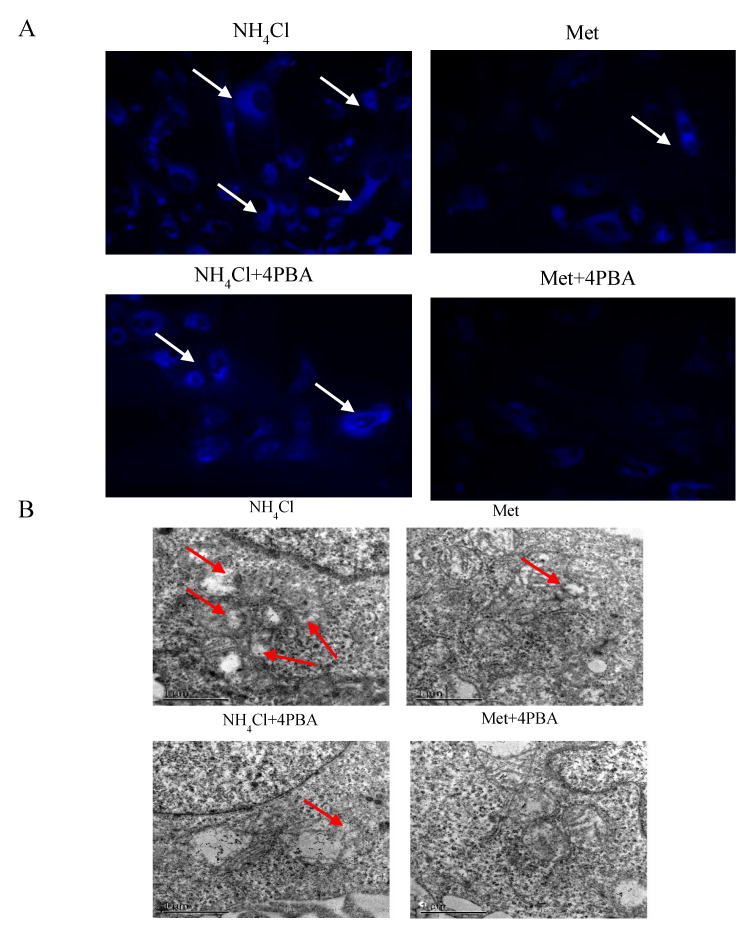

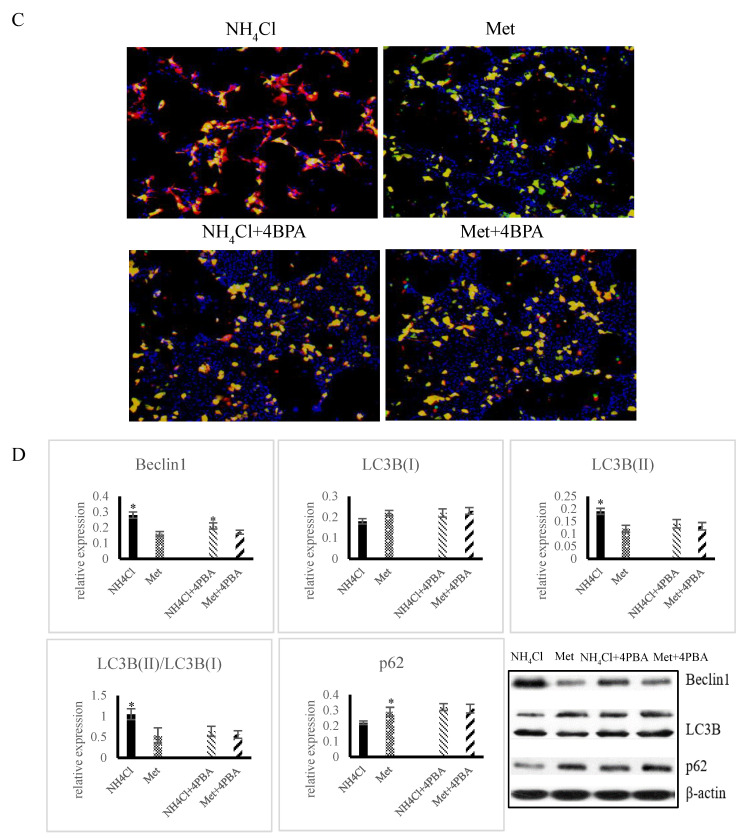
Effects of ER stress in NH_4_Cl and Met-induced autophagy in PRECs. (**A**) Autophagic morphological changes were observed by fluorescent microscopy using MDC staining; the images were viewed under a 200× fluorescence microscope. (**B**) The effect of 4PBA on autolysosomes number in PRECs measured by double staining with 3% uranium acetate and lead nitrate. The images were viewed under a 40,000× transmission electron microscope. (**C**) Immunofluorescence image of endogenous LC3 in PREC cultured treated with NH_4_Cl and Met. Green dots represent autophagosomes, red dots represent autolysosomes. (**D**) The effect of 4PBA on relative expression of autophagic proteins was measured by Western blot. Each bar indicates mean ± SD. Compared with the control, * *p* < 0.05 shows the difference is statistically significant.

There were significantly fewer (*p* < 0.05) autophagosomes in NH_4_Cl + 4PBA-treated cells than in NH_4_Cl-treated cells, while no significant differences (*p* > 0.05) were observed between Met + 4PBA-treated cells and Met-treated cells (Figure 5A,B). After GFP-LC3 plasmid transfection, there were fewer LC3 spots in the NH_4_Cl + 4PBA group (*p* < 0.05) than in the NH_4_Cl group (Figure 5C). The protein expression levels of Beclin1 and LC3-II were lower, while those of p62 were significantly higher (*p* < 0.05), in the NH_4_Cl + 4PBA group in comparison with the NH_4_Cl group (Figure 5D). The protein expression levels of Beclin1, LC3-II, and p62 in the Met + 4PBA group were not significantly different from those in the Met group (*p* > 0.05).

### 3.5. Crosstalk between Apoptosis and Autophagy Induced by NH_4_Cl and Met in PRECs

**Figure 6:**
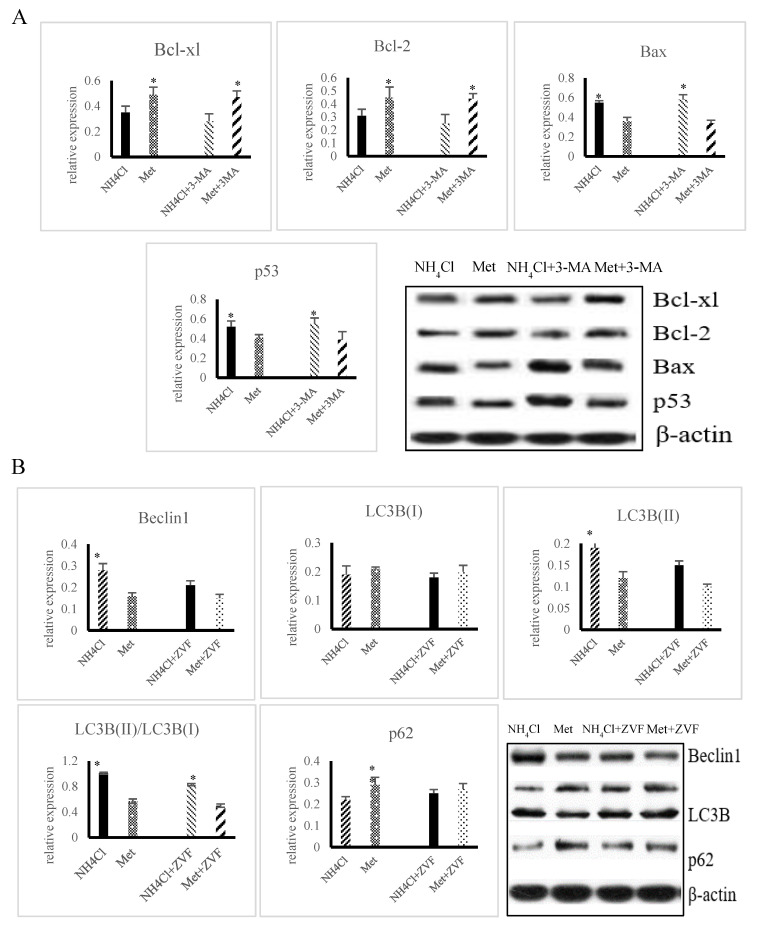
Crosstalk between apoptosis and autophagy induced by NH_4_Cl and Met in PRECs. (**A**) The role of 3-MA in apoptosis induced by NH_4_Cl and Met was confirmed by Western blot. Each bar shows mean ± SD. (**B**) The role of Z-VAD-FMK (ZVF) in autophagy by NH_4_Cl and Met was measured by Western blot.

The relative protein expression of Bax and caspase-3 was significantly higher (*p* < 0.05), while that of Bcl-2 and Bcl-xl was significantly lower (*p* < 0.05) in the NH_4_Cl + 3-MA(3-Methyladenine) group than in the NH_4_Cl group. In addition, the relative protein expression of Bax and caspase-3 was significantly lower (*p* < 0.05), while that of Bcl-2 and Bcl-xl was significantly higher (*p* < 0.05), in the Met + 3-MA group in comparison with the NH_4_Cl group (Figure 6A). As shown in Figure 6B, the relative protein expression of Beclin1 and LC3B-II was significantly lower (*p* < 0.05), while that of p62 was significantly higher (*p* < 0.05), in the NH_4_Cl + Z-VAD-FMK group in comparison with the NH_4_Cl group, while no significant difference was observed between the Met + Z-VAD-FMK group and the Met group (*p* > 0.05). The protein expression of Beclin1 and LC3-II was significantly higher (*p* < 0.05), while that of p62 was significantly lower (*p* < 0.05), in the NH_4_Cl group in comparison with the Met + Z-VAD-FMK group.

Western blot analysis was performed in order to determine the effect of on autophagy induced for 36 h. Each bar shows mean ± SD.

## 4. Discussion

In ruminants, the self-renewal process of rumen epithelial cells is affected by more factors than that of other types of cells because the rumen is in direct contact with the diet. During self-renewal, the mucosal epithelial cells of the gastrointestinal tissue need to continuously synthesize DNA, RNA, and proteins, and thus proteins from various sources are very important for the growth, regulation, and repair of gastrointestinal mucosal epithelial cells [23,24]. In addition, the regeneration and repair of epithelial cells are inseparable from apoptosis and autophagy, which frequently occur in epithelial cells. Therefore, studying the relationships between different nitrogen sources and apoptosis/autophagy is critical.

Apoptosis is an important mechanism of NH_4_Cl toxicity, and ROS contribute substantially to NH_4_Cl-induced apoptosis [10]. High levels of NH_4_Cl promote ROS production, mitochondrial dysfunction, and cytochrome c release, and eventually lead to apoptosis [11]. In the present work, the levels of both ROS and p53 were increased in NH_4_Cl-treated cells. We hypothesized that ROS activated p53-regulated mitochondrial apoptosis pathways during NH_4_Cl-induced apoptosis. ROS concentrations are correlated with the disruption of mitochondrial membrane potential Δψm [25], which is consistent with the results regarding MMP obtained in the present work—the NH_4_Cl group exhibited higher ROS levels than the Met group. When apoptosis occurs, the p53-regulated Bcl-2 protein family (including Bax and Bcl2) activates the mitochondrial apoptotic pathway [26]. In the current study, changes in the relative protein expression of p53 and Bax were positively correlated with changes in the apoptosis rate. p53 can disrupt the Bax/Bcl-2 balance by interacting with Bcl-2 protein in the mitochondria, increasing the permeability of the outer mitochondrial membrane [27]; this phenomenon is consistent with our findings that p53 levels were higher, while the MMP and Bax/Bcl-2 ratio was lower, in the NH_4_Cl group in comparison with the Met group. Therefore, we conclude that NH_4_Cl induces apoptosis by causing mitochondrial dysfunction.

Autophagy is a lysosomal-dependent degradation pathway and a dynamic process, the last step of which is the fusion of autophagosomes and lysosomes to degrade cytoplasmic substances and organelles [28]. Previous studies have reported that Met deficiency leads to autophagy of glioma cells both in vitro and in vivo [29], consistent with our findings that the levels of autophagy-related proteins and the numbers of autophagosomes were higher in NH_4_Cl-treated cells than in Met-treated cells. In this study, we observed significantly increased LC3-II/LC3-I ratios and decreased p62 levels in NH_4_Cl-treated cells, which might have resulted from the fact that there is no colocalization between mitochondria and lysosomes when cells are treated with NH_4_Cl under starvation conditions, which leads to decreased autophagy [14]. Therefore, we speculate that autophagy in the NH_4_Cl group was induced by ER stress.

A previous study has revealed that NH_4_Cl induces apoptosis and autophagy through ER stress and mitochondrial dysfunction in MRP2 cells [11]. Thus, we aimed to explore the relationship between ER stress and apoptosis/autophagy induced by NH_4_Cl in this study. Pretreatment with 4PBA reduced NH_4_Cl-mediated induction of apoptosis and autophagy, implying that ER stress helps regulate apoptosis and autophagy in PRECs. In the present work, the levels of CHOP increased with upregulation of Bax and p53 expression in NH_4_Cl-treated cells, perhaps because ER stress activated CHOP; triggered Bcl-2, Bak, and Bax expression; and ultimately activated ROS-induced apoptosis [30]. Additionally, a previous study has found that PC12 cells might be injured by ER stress-mediated apoptosis [31]. Furthermore, activation of ER stress pathways has also been reported to be associated with autophagy [32,33]. In the present study, we observed significant increase in LC3-II and a decrease in p62 with increased CHOP expression in NH_4_Cl-treated cells, indicating that the increase in autophagy was associated with the activation of ER stress. In conclusion, ER stress contributes to the apoptosis and autophagy triggered by NPN in PRECs, perhaps because that NPN can promote the occurrence of oxidative stress [34].

Apoptosis and autophagy can occur simultaneously in Bel-7402 cells, and autophagy can regulate apoptosis via phosphorylation of p38 MAPK. However, autophagy promotes cell proliferation and survival in response to stress and inhibits apoptosis [35,36]. In this study, we found that downregulation of autophagy via treatment with 3-MA significantly intensified apoptosis triggered by oxidative stress in NH_4_Cl-treated cells; conversely, inhibition of apoptosis with Z-VAD-FMK alleviated autophagy in NH_4_Cl-treated cells. However, we did not find a similar phenomenon in cells treated with Met. Thus, we suggest that autophagy might have a palliative effect on apoptosis in cells treated with NPN compared with protein nitrogen, possibly because that protein nitrogen can better provide the carbon skeleton and energy needed for cellular protein synthesis and can promote cell growth.

## 5. Conclusions

Our results suggest that ER stress contributes substantially to the apoptosis and autophagy triggered by protein nitrogen and NPN. Autophagy has a more obvious ameliorative effect on apoptosis in ruminal epithelial cells treated with NPN than in ruminal epithelial cells treated with protein nitrogen, perhaps because protein nitrogen better suited than NPN for protein synthesis and promotes the resistance of cells to AA deficiency stress.

## Figures and Tables

**Table 1:** Gene primers.

Gene Name	GeneBank No.	Primer Sequence (5′-3′)	Product Size (bp)
		CTGAGGGATGGAAGGGTC	
Beclin1	XM_017025264		159
		TGGGCTGTGGTAAGTAATG	
		CGGGTTGAGGAGACACACAA	
LC3B	XM_011529083		141
		ATGAG CCGGACATCTTCCAC	
		CCATGCAGGTGAGCTTCGT	
p62	XM_018051607		155
		GAATCTGCGAGAGACACCATC	
		GCTACGACACGGAGTTCCA	
Bcl-2	XM_005696234		113
		CCCAGTTGATGCCGCTCT	
		GAGCTGGTTGACTTTCTC	
BCL-XL	XM_006498612		
		TCCATCTCCGATTCAGTCCCT	142
		TGAAGCGCATTGGAGATG	
Bax	XM_013971446.2		185
		GGCCTTGAGCACCAGTTT	
		GAATGTCCGAATGAAGCG	
p53	XM_005693530		121
		CGTAGTTGCCAGGGTAGG	
		ATGGCTACTGCTGCGTCGT	
β-actin	AF481159·1		161
		TTGAAGGTGGTCTCGTGGAT	

**Table 2:** Details of the primary antibodies.

Name	No.	Host Species	Dilution	Supplier
Caspase-3	9665s	Rabbit	1:1000	CST
BCL-2	ab59348	Rabbit	1:1000	Abcam
BAX	50599-2-AP	Rabbit	1:2000	Proteintech
P53	10442-1-AP	Rabbit	1:1200	Proteintech
BCL-XL	10783-1-AP	Rabbit	1:1200	Proteintech
LC3	ab192890	Rabbit	1:2000	Abcam
Beclin1	ab62557	Rabbit	1:1500	Abcam
p62	ab155686	Rabbit	1:2000	Abcam
DAPK1	25136-1-AP	Rabbit	1:1000	Proteintech
GPR78	ab198787	Rabbit	1:1000	Abcam
ATF4	ab184909	Rabbit	1:1000	Abcam
CHOP	ab179823	Rabbit	1:2000	Abcam
β-actin	ab179467	Rabbit	1:5000	Abcam
